# Supplementary material for: Elongation inhibitors do not prevent the release of puromycylated nascent polypeptide chains from ribosomes
Source: eLife. 2020 Aug 26;9:e60048. doi: 10.7554/eLife.60048 (PMC7490010; doi:10.7554/eLife.60048)
Supplement: Figure 1—source data 1. — Summary of replicates, cells, and statistical testing (Mann-Whitney U) for indicated treatment groups. [file elife-60048-fig1-data1.docx]

**Figure 1 – source data 1**

Statistical information pertaining to Figure 1C and Figure 1 – Supplementary Figure 2C

| **Treatment** | **Replicates** | **Cells** | **Median** | **Mean** | **StDev** |  |  |
| --- | --- | --- | --- | --- | --- | --- | --- |
| mRG.NoPuro_IF | 4 | 288 | 0.068 | 0.074 | 0.062 |  |  |
| mRG.NoPuro_PLA | 4 | 144 | 0.068 | 0.060 | 0.076 |  |  |
| mRG.Puro_IF | 4 | 288 | 0.930 | 0.972 | 0.433 |  |  |
| mRG.Puro_PLA | 4 | 144 | 0.886 | 0.924 | 0.441 |  |  |
| mRG.EmtPuro_IF | 4 | 288 | 0.942 | 0.969 | 0.383 |  |  |
| mRG.EmtPuro_PLA | 4 | 144 | 0.780 | 0.821 | 0.408 |  |  |
| mRG.EmtPuroNoHA_IF | 3 | 144 | 0.862 | 0.883 | 0.357 |  |  |
| mRG.EmtPuroNoHA_PLA | 3 | 112 | 0.052 | 0.055 | 0.048 |  |  |
| mRG.EmtPuroNoPuro_IF | 3 | 144 | 0.073 | 0.074 | 0.024 |  |  |
| mRG.EmtPuroNoPuro_PLA | 3 | 112 | 0.099 | 0.097 | 0.084 |  |  |
| mRG.AnisoPuro_IF | 4 | 288 | 0.122 | 0.136 | 0.107 |  |  |
| mRG.AnisoPuro_PLA | 3 | 112 | 0.057 | 0.055 | 0.059 |  |  |
| U87.EmtPuro_IF | 3 | 264 | 0.844 | 0.875 | 0.388 |  |  |
| U87.EmtPuro_PLA | 3 | 112 | 0.150 | 0.162 | 0.138 |  |  |
| mRG.PuroWash15_IF | 3 | 128 | 0.906 | 0.970 | 0.510 |  |  |
| mRG.PuroWash15_PLA | 3 | 128 | 0.942 | 0.980 | 0.460 |  |  |
| mRG.PuroWash45_IF | 3 | 96 | 1.081 | 1.105 | 0.436 |  |  |
| mRG.PuroWash45_PLA | 3 | 96 | 0.910 | 0.970 | 0.569 |  |  |
| mRG.HarrPuro_IF | 3 | 96 | 0.095 | 0.096 | 0.035 |  |  |
| mRG.HarrPuro_PLA | 3 | 96 | 0.114 | 0.118 | 0.052 |  |  |
| mRG.PuroWashHarr_IF | 3 | 96 | 0.746 | 0.795 | 0.333 |  |  |
| mRG.PuroWashHarr_PLA | 3 | 96 | 0.619 | 0.640 | 0.342 |  |  |
|  |  |  |  |  |  |  |  |
| **Group1** | **Group2** | **Median**  **Group1** | **Median**  **Group2** | **U** | **p** | **n_1_** | **n_2_** |
| mRG.Puro_IF | mRG.EmtPuro_IF | 0.930 | 0.942 | 41096 | 0.4254 | 288 | 288 |
| mRG.Puro_PLA | mRG.EmtPuro_PLA | 0.886 | 0.780 | 8739 | 0.0106 | 144 | 144 |
| mRG.Puro_IF | mRG.Puro_PLA | 0.930 | 0.886 | 18997 | 0.0776 | 288 | 144 |
| mRG.EmtPuro_IF | mRG.EmtPuro_PLA | 0.942 | 0.780 | 15544 | 1.10E-05 | 288 | 144 |
| mRG.EmtPuroNoHA_IF | mRG.EmtPuroNoHA_PLA | 0.862 | 0.052 | 15 | 5.47E-43 | 144 | 112 |
| U87.EmtPuro_IF | U87.EmtPuro_PLA | 0.844 | 0.150 | 454 | 2.67E-50 | 264 | 112 |
| mRG.PuroWash15_IF | mRG.PuroWash15_PLA | 0.906 | 0.942 | 7736 | 0.220959 | 128 | 128 |
| mRG.PuroWash45_IF | mRG.PuroWash45_PLA | 1.081 | 0.910 | 3442 | 0.001233 | 96 | 96 |
| mRG.PuroWashHarr_IF | mRG.PuroWashHarr_PLA | 0.746 | 0.619 | 3200 | 1.28E-04 | 96 | 96 |

Summary of replicates, cells, and statistical testing (Mann-Whitney U) for indicated treatment groups.
