# Supplementary material for: Elongation inhibitors do not prevent the release of puromycylated nascent polypeptide chains from ribosomes
Source: eLife. 2020 Aug 26;9:e60048. doi: 10.7554/eLife.60048 (PMC7490010; doi:10.7554/eLife.60048)

<u>Fig2A Blots</u>

Emt + Puro

<u>Fig2B Blots</u>

Chx + Puro

1st probes

Rb anti-S6 / Ms anti-HA

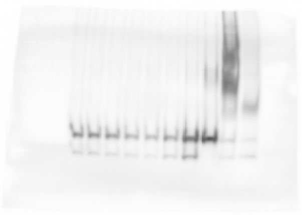

Ms anti-Puro

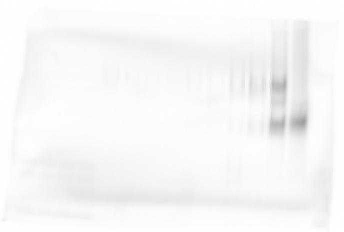

2nd probes

Ms anti-Puro

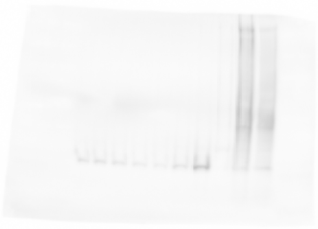

Rb anti-S6 / Ms anti-HA

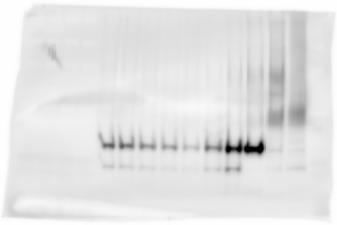

<u>Fig2C Blots</u>

Ms anti-HA

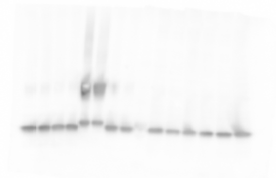

Rb anti-S6

lower exposure

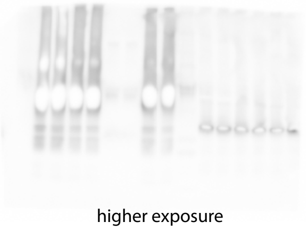

higher exposure

Ms anti-Puro

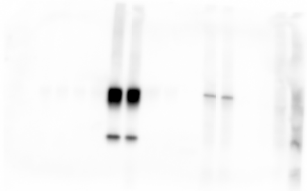

Supplement: Figure 2—source data 1. [file elife-60048-fig2-data1.pdf]
