# Supplementary material for: Elongation inhibitors do not prevent the release of puromycylated nascent polypeptide chains from ribosomes
Source: eLife. 2020 Aug 26;9:e60048. doi: 10.7554/eLife.60048 (PMC7490010; doi:10.7554/eLife.60048)
Supplement: Figure 4—source data 1. [file elife-60048-fig4-data1.docx]

**Figure 4 – source data 1**

Summary of MolProbity analysis of IgG2a Fab alignments within 80S A site

| **Model #** | **MolProbity Clashscore^a^** | **Structures of Comparable Resolution With Worse MolProbity Clashscores (%)^b^** |  |
| --- | --- | --- | --- |
| 1 | 246.30 | 0 |  |
| 2 | 196.19 | 0 |  |
| 3 | 183.82 | 0 |  |
| 4 | 177.55 | 0 |  |
| 5 | 155.58 | 0 |  |
| 6 | 143.09 | 0 |  |
| 7 | 134.19 | 0 |  |
| 8 | 126.94 | 0 |  |
| 9 | 107.07 | 0 |  |
| 10 | 105.83 | 0 |  |
| 11 | 91.18 | 0 |  |
| 12 | 85.79 | 0 |  |
| Best Fit in A Site | 22.89 | 25 |  |
| **Structure** | **PDB ID** | **MolProbity Clashscore^a^** | **Structures of Comparable Resolution With Worse MolProbity Clashscores (%)** |
| Ms IgG2a | 1IGT | 20.63 | 87**^c^** |
| Rb 80S | 6SGC | 7 | 87^b^ |

^a^ The number of serious steric overlaps (> 0.4 Å) per 1000 atoms

^b^ Out of 1,784 structures solved using cryo-EM at any resolution

^c^ Out of 141 structures solved with X-ray crystallography with resolutions of 2.80 ± 0.25 Å
