# Supplementary material for: Elongation inhibitors do not prevent the release of puromycylated nascent polypeptide chains from ribosomes
Source: eLife. 2020 Aug 26;9:e60048. doi: 10.7554/eLife.60048 (PMC7490010; doi:10.7554/eLife.60048)
Supplement: Transparent reporting form [file elife-60048-transrepform.docx]

***eLife’s* transparent reporting form**

We encourage authors to provide detailed information *within their submission* to facilitate the interpretation and replication of experiments. Authors can upload supporting documentation to indicate the use of appropriate reporting guidelines for health-related research (see [EQUATOR Network](http://www.equator-network.org/%20)), life science research (see the [BioSharing Information Resource](https://biosharing.org/)), or the [ARRIVE guidelines](http://www.plosbiology.org/article/info:doi/10.1371/journal.pbio.1000412) for reporting work involving animal research. Where applicable, authors should refer to any relevant reporting standards documents in this form.

If you have any questions, please consult our Journal Policies and/or contact us: [editorial@elifesciences.org](mailto:editorial@elifesciences.org).

**Sample-size estimation**

- You should state whether an appropriate sample size was computed when the study was being designed
- You should state the statistical method of sample size computation and any required assumptions
- If no explicit power analysis was used, you should describe how you decided what sample (replicate) size (number) to use

Please outline where this information can be found within the submission (e.g., sections or figure legends), or explain why this information doesn’t apply to your submission:

As the development of a puromycin/eL22-HA proximity ligation assay was an exploratory endeavor, we did not initially compute sample sizes. Based on initial results and antibody omission controls, we determined that 3-4 separate experimental replicates, each with 36-72 cells quantified, would be sufficient for statistical analysis.

Similarly, based on preliminary observations with SunTag live cell imaging, we found that 3-4 separate replicate imaging trials, with 7-12 cells each, enabled accurate kinetic analysis presented in Figure 3: see Figure 3 – figure supplement 1 for reproducibility of replicate imaging trials.

For structural modeling presented in Fig. 4, there is no basis for sample size estimation when constructing rigid body docking models of Fab fragments contacting puromycin in the PTC. We chose to evaluate 12 models in order to cover the full range of angles and positions that the Fab could realistically fit in within the A site (see Methods section).

**Replicates**

- You should report how often each experiment was performed
- You should include a definition of biological versus technical replication
- The data obtained should be provided and sufficient information should be provided to indicate the number of independent biological and/or technical replicates
- If you encountered any outliers, you should describe how these were handled
- Criteria for exclusion/inclusion of data should be clearly stated
- High-throughput sequence data should be uploaded before submission, with a private link for reviewers provided (these are available from both GEO and ArrayExpress)

Please outline where this information can be found within the submission (e.g., sections or figure legends), or explain why this information doesn’t apply to your submission:

The table presented in Figure 1 – source data 1 shows all replicates, sample sizes, and statistical information pertinent to Figure 1 and associated figure supplements.

For Figure 2 – Figure Supplement 1, Figure 3, and Figure 4, replicate details and statistical information are present in the figure caption as well as in the methods section.

**Statistical reporting**

- Statistical analysis methods should be described and justified
- Raw data should be presented in figures whenever informative to do so (typically when N per group is less than 10)
- For each experiment, you should identify the statistical tests used, exact values of N, definitions of center, methods of multiple test correction, and dispersion and precision measures (e.g., mean, median, SD, SEM, confidence intervals; and, for the major substantive results, a measure of effect size (e.g., Pearson's r, Cohen's d)
- Report exact p-values wherever possible alongside the summary statistics and 95% confidence intervals. These should be reported for all key questions and not only when the p-value is less than 0.05.

Please outline where this information can be found within the submission (e.g., sections or figure legends), or explain why this information doesn’t apply to your submission:

Details regarding replicates, cell numbers, and test statistics are provided in the figure captions (except see Figure 1 – source data 1 for summary of testing presented in Figure 1C and Figure 1 – figure supplement 2C).

A summary of statistical tests employed is also present in the ‘Statistical Analysis’ section of the Materials and Methods.

(For large datasets, or papers with a very large number of statistical tests, you may upload a single table file with tests, Ns, etc., with reference to sections in the manuscript.)

**Group allocation**

- Indicate how samples were allocated into experimental groups (in the case of clinical studies, please specify allocation to treatment method); if randomization was used, please also state if restricted randomization was applied
- Indicate if masking was used during group allocation, data collection and/or data analysis

Please outline where this information can be found within the submission (e.g., sections or figure legends), or explain why this information doesn’t apply to your submission:

This information does not pertain to our submission, since all experimental groups were based on distinct drug treatments in wells of identically cultured cells.

**Additional data files (“source data”)**

- We encourage you to upload relevant additional data files, such as numerical data that are represented as a graph in a figure, or as a summary table
- Where provided, these should be in the most useful format, and they can be uploaded as “Source data” files linked to a main figure or table
- Include model definition files including the full list of parameters used
- Include code used for data analysis (e.g., R, MatLab)
- Avoid stating that data files are “available upon request”

Please indicate the figures or tables for which source data files have been provided:

We have included source data (both numerical and raw images of Western blots) for Figure 1C, Figure 1- Figure Supplement 1B, Figure 1 – Figure Supplement 2C, Figure 2A-C, Figure 2 – Figure Supplement 1A, Figure 3C-D, and Figure 4B.
